# Comparison of breastfeeding self-efficacy and breastfeeding success of obese and normal-weight mothers in the early period

**DOI:** 10.4314/ahs.v20i4.60

**Published:** 2020-12

**Authors:** Sinem Ceylan, Sevil Şahin

**Affiliations:** 1 Ankara Medipol University, Ankara, Turkey ceylansinem1@gmail.com; 2 Ankara Yildirim Beyazit University, Faculty of Health Sciences, Ankara, Turkey sevilsahin1@gmail.com

**Keywords:** Breastfeeding success, self-efficacy, maternal obesity

## Abstract

**Background:**

Body mass index (BMI) of overweight and obese women is a risk factor for breast milk secretion.

**Aim:**

This study was conducted in a descriptive and comparative way in order to identify the relation between the breastfeeding success and self-efficacy of obese and non-obese mothers during postnatal period and to make a comparison between the obese and non-obese group.

**Methods:**

The study sample consisted of 113 obese and 111 non-obese mothers that met the study criteria who were hospitalized at the postnatal service of Health Education and Research Hospital in Ankara, September 2014-February 2015.

**Result:**

The mean BMI of obese women prior to the pregnancy was 31.41±2.4 while it was 22.25±2.8 for the non-obese women. As the BSS scores increase among both the obese and non-obese mothers, the LATCH breastfeeding success score averages increase as well (p<0.05, r:0.613).

**Conclusion:**

In the light of the data of, to increase the breastfeeding success among obese mothers, it is suggested that the perception of self-efficacy is enhanced, and additional consultation is provided on breastfeeding starting from the antenatal period. The trainings given to mothers by the midwife and nurse are supported with home visits especially in the obese women in the postnatal period in order for them to breastfeeding.

## Introduction

Breastfeeding is known to reduce mortality and morbidity rates in infants, to ensure appropriate nutrition, growth and development for infants and to provide economic benefits to family and country with proven superiority to other forms of feeding. Breast milk is the ideal food for the infancy due to several reasons such as varying contents based on infant's needs, features protecting from infections, meeting infant's physiological and psycho-social requirements alone in the first six months and economic advantages 1, 2. In several studies, the mother's educational status, lack of home-care support for the mother and smoking were identified as some of the factors affecting exclusive breastfeeding during the first six months 3, 4, 5. Furthermore, there is a negative relationship between maternal obesity and breastfeeding 6, 7.

The prevalence of obesity in pregnancy varies from 1.8% to 25.3% according to the WHO 8. Studies indicate that the prevalence of obesity among pregnant women is 28.9%, a trend that continues to rise 9. Obesity is an important risk factor affecting postnatal breastfeeding^6^. There are physiological and psycho-social mechanisms affecting breastfeeding in obese women. Studies showed that maternal obesity (BMI greater than 30 kg/m^2^) has negative effects on breastfeeding initiation and duration. BMI of overweight and obese women is a risk factor for breast milk secretion. As the prolactin level is lower in breast milk of obese women, milk production decrases. Newborns may have difficulty in sucking due to some biological factors such as large breasts, large areolae or inverted nipples in overweight and obese women 10.

Overweight women have less tendency to initiate and maintain breastfeeding psychologically. Such women are reported to have a negative self-image and low self-confidence as a result of many factors including mental health problems and postnatal depression. Furthermore, overweight women may avoid breastfeeding because of having large breasts from behavioral perspective and lactation may be shorter due to mechanical difficulties in holding and supporting the baby and reduced levels of prolactin in large-breasted women 11. There are a wide range of factors affecting exclusive breastfeeding at levels lower than desired. The most important one is mothers' lack of information on exclusive breastfeeding, maintenance of breastfeeding, coping with barriers and ideal time to initiate complementary feeding. Accordingly, it is clear that training provided by nurses in the antenatal and postnatal period will support breastfeeding. Nurses can support mothers in deciding to, initiation and maintain of breastfeeding and provide guidance on the ideal time to initiate complementary feeds^3^.

This study was conducted to compare self-efficacy and successful breastfeeding in the early postpartum period among obese women and normal-weight women during hospitalization period.

## Method

### Type of Study

This is a descriptive and comparative study.

### Study population

The study population consisted of 8691 delivered women who presented to Zekai Tahir Burak Hospital from 15^th^ September, 2014 to 15^th^ February, 2015. Antenatal BMI was taken as ≥30 in obese group. Women with a BMI of <18.5 were excluded.

### Sample of the Study

The sample size was 105 for each group in the power analysis where alpha was 0.05, beta was 0.95 and the effect size was 0.05. Of the women who presented to Zekai Tahir Burak Hospital from 15/09/2014 to 15/02/2015, were requested to voluntarily take part in the study; completed a consent form and delivered a baby. One hundred and thirteen obese and 111 normal-weight women were included in the study. Inclusion criteria for the study included having a prenatal BMI of ≥30 kg/m^2^ in obese group and <18.5 kg/m2 in normal-weight group, having no chronic diseases, having been delivered at least 24 hours ago a full-term and healthy single baby and performing breastfeeding.

### Ethical aspects of the study

Written approvals were obtained from the Ethics Committee of Yildirim Beyazit University to conduct the study. Permission was obtained from the Education Planning Committee of Zekai Tahir Burak Women Health Training and Research Hospital where the study was conducted. The study was supported by the Scientific Research Projects Unit of Yildirim Beyazit University with the project number 1670. Before the questionnaire was administered, the participants were informed about the purpose of the study and contents of the form and asked to read and sign the consent form for their participation. The participants were informed about the fact that personal data used on the consent form and inhe study would be kept confidential and that they could withdraw from the study any time if they so wished. This would not affect their care.

### Data collection instruments

The Personal Information Form prepared by the investigator in line with the literature 3, 5, 12 on demographic characteristics of the mother and infant, Breastfeeding Self-Efficacy Scale and the LATCH Breastfeeding Charting and Documentation Tool 13,14 were used as data collection instruments.

### Personal Information Form

The forms consists of four sections, namely mother details, pregnancy details, infant details and breastfeeding details. This form questioned mother's details (mother's age, education, working status, family's income level, body mass index before and at the end of pregnancy), pregnancy details (intended pregnancy, number of children, mode of delivery), infant's details (gender, gestational week) and breastfeeding details (breastfeeding experience, having breastfeeding training, breastfeeding initiation time, how long to continue breastfeeding).

### Breastfeeding Self-Efficacy Scale-Short Form (Postnatal Version)

The original 33-item scale was developed by Dennis and Faux to assess maternal self-efficacy levels. In 2003, 14-item short form of the scale was developed. Dennis suggests the use of this short form 13. As recommended by Bandura, all items are presented positively. This is a 5-point Likert-type scale where 1 indicates “not at all confident” and 5 indicates “always confident”. Scores are summed to produce a range from 14 to 70, with higher scores indicating higher levels of breastfeeding self-efficacy. Turkish reliability and validity study of the scale was performed by Tokat and Cronbach's Alpha value was determined as 0.86 13, 14. Cronbach's Alpha value of the scale was determined as 0.97 in our study.

### LATCH Breastfeeding Charting and Documentation Tool

The LATCH charting tool contains five key assessment components to which a numerical score of 0, 1, or 2 is assigned for a possible total score of 10 points. Higher scores indicate high breastfeeding success. Turkish validity of the documentation tool was performed by Demirhan in 1997, Koyun in 2001, and Yenal and Okumus in 2003 and it was suggested as a reliable tool. Cronbach's Alpha value of the LATCH Breastfeeding Charting and Documentation Tool was determined to be 0.95 by Yenal and Okumus, 0.94 by Demirhan and 0.96 by Koyun 15, 16, 17. Cronbach's Alpha value of the LATCH tool was determined as 0.76 in our study. The LATCH tool was assessed in our study by observing one breastfeeding session of mothers participating in our study.

### Conduct of the study

A pilot study was conducted on 15 women eligible for the study criteria to determine intelligibility of data collection forms (“Personal Information Form”, “Breastfeeding Self-Efficacy Scale”, “LATCH” Breastfeeding Charting and Documentation Tool”) and change of perception during implementation. After the pilot study, questions presenting difficulty in understanding were revised. Data obtained was not included in the analysis.

In this stage, data collection forms were administered by the same investigator face to face to mothers who were eligible for the study criteria and agreed to take part in the study. Data collection period was flexible for each mother who agreed to take part in the study and it was continued to collect data on a daily basis within working hours until the sample size was reached. Interviews were conducted in separate rooms to prevent interaction of mothers.

### Data Assessment

The study consisted of 224 data sets. Data was integrated by transferring to IBM SPSS Statistics 22.0 package program. Descriptive analyses (mean ± standard deviation) were used for digital data and frequency distribution was given for categorical variables while assessing the study data. The Independent Samples T-Test was used to determine whether there was any difference between two independent groups. One-way Analysis of Variance was used to determine the difference between more than two independent groups. The association between two categorical variables was measured with the Chi-square test at equal interval level and the Pearson's correlation analysis was used to examine the association between variables. The Kolmogorov-Smirnov normality test was used to examine normality for continuous variables. The significance level was accepted as p<0.05 in the study.

## Results

In this study, mean age was 27.05±6.1 and 25.19±5.1 for obese and non-obese mothers, respectively. Considering the preconception body mass index of mothers in the study, preconception BMI was determined as 31.41±2.4 in obese mothers and as 22.25±2.8 in normal-weight mothers. Distribution of individual maternal characteristics is given in Table 1.

**Table 1:** Distribution of Individual Characteristics of Obese and Normal-Weight Mothers

Individual Characteristics			Obese (n=113) X±SD		Non-obese (n=111) X±SD		t	p
**Age**			27.05±6.1		25.19±5.1		2.470	0.014*

BMI of mother before pregnancy***			31.41±2.4		22.25±2.8		25.503	0.000**

	**Obese**		**Non-obese**		**Total**		**X^2^**	**p**
	**n**	**%**	**n**	**%**	**n**	**%**		

**Level of Education**								
Primary education	82	72.6	86	77.5	168	75.0	1.911	0.385
High school	28	24.8	20	18.0	48	21.4		
University	3	2.7	5	4.5	8	3.6		

**Working Status**								
Yes	6	5.3	4	3.6	10	4.5	0.382	0.385
No	107	94.7	107	96.4	214	95.5		

**Profession**								
Civil Servant/Worker	4	66.7	2	33.3	6	50.0	0.333	0.567
Tradesman/Self-employed	2	33.3	4	66.7	6	50.0		

**Income Status**								
Income less than expenses	14	12.5	15	13.5	29	13.0	0.985	0.611
Equal income and expense	94	83.9	89	80.2	183	82.1		
Income more than expenses	4	3.6	7	6.3	11	4.9		

*:*p<0.05* ***BMI: Body Mass Index

t: Independent Samples T-test, X^2^: Chi-square test

Although not listed in the table, based on the number of childbirth, number of pregnancy, mode of delivery and regular follow-up during pregnancy for the mothers who participated in the study, it was determined that the both groups were similar with no statistically significant difference.

There was no statistically significant relationship between obesity of mothers in the study, whether they received breast milk training and first breastfeeding time after the delivery (p>0.05). It was determined that mothers who were trained on breast milk in both groups received training/information at healthcare institutions mostly. A comparison of breastfeeding characteristics of obese and normal-weight mothers is given in Table 2.

**Table 2:** Comparison of Breastfeeding Characteristics of Obese and Normal-Weight Mothers

	Obese		Non-obese		Total		X2	p
	n	%	n	%	n	%		
**Obtaining information on breast milk**								
Yes	39	34.5	30	27.0	69	30.8	1.472	0.225
No	74	65.5	81	73.0	155	69.2

**Sources of information**								
Health Institution	21	53.8	23	76.7	44	63.8		
Internet	1	2.6	0	0.0	1	1.4	-	-
Pregnancy Class	2	5.1	1	3.3	3	4.3		
Relatives-Acquaintances	15	38.5	6	20.0	31	30.4		

**First breastfeeding after delivery**								
Within first 1 hour	112	99.1	108	97.3	220	98.2	1.055	0.304
After 1 hour	1	0.9	3	2.7	4	1.8		

**Breastfeeding interval**								
1–2 hours	43	38.1	29	26.1	72	32.1		
3–4 hours	5	4.4	1	0.9	6	2.7	-	-
Whenever the infant cries	65	57.5	81	73.0	146	65.2		

**Breastfeeding duration**								
5 min.	3	2.7	3	2.7	6	2.7		
6–10 min.	17	15.0	10	9.0	27	12.1		
11–15 min.	32	28.3	25	22.5	57	25.4	-	-
16–20 min.	39	34.5	43	38.7	82	36.6		
20 min.↑	22	19.5	30	27.0	52	53.2		

**Use of supplementary food other than** **breast milk**								
Yes	41	36.3	22	19.8	63	28.1	7.508	0.006
No	72	63.7	89	80.2	161	71.9		*

**Liquid/food type given**								
Water	1	2.4	1	4.5	2	3.1	-	-
Formula	40	97.6	21	95.5	61	96.9		

*:*p<0.01* t: Independent Samples T-test, X^2^: Chi-square test

**Figure 1:**
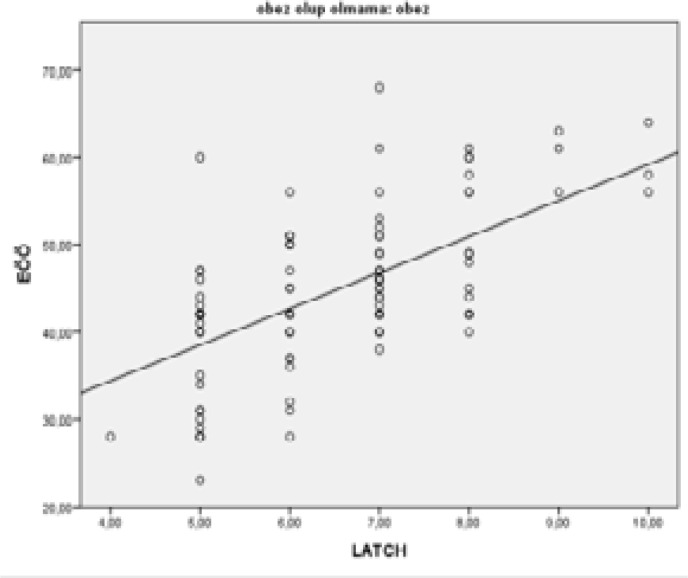
Relationship between postnatal breastfeeding self-efficacy score and LATCH breastfeeding success score of obese mothers.

**Figure 2:**
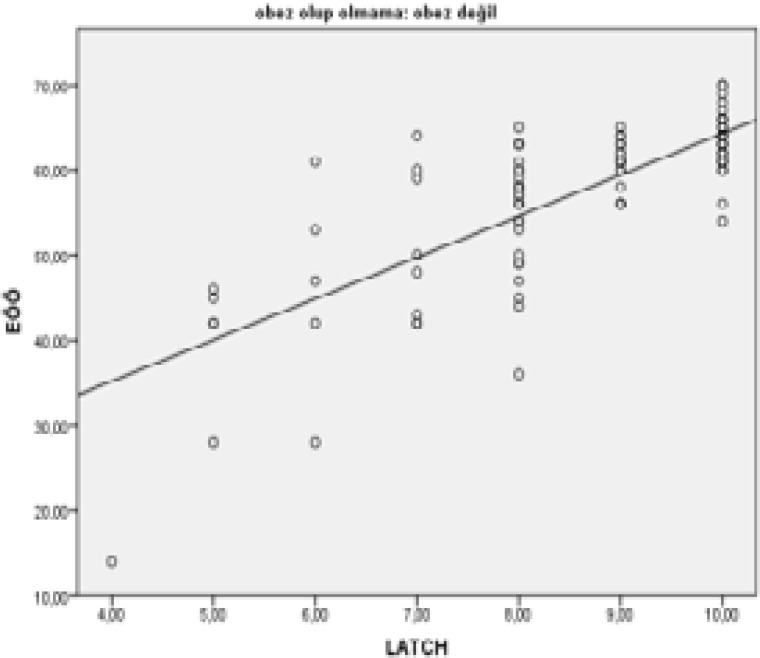
Breastfeeding self-efficacy and LATCH scores of normal-weight mothers in postnatal period

Upon examination of Table 3, Figure 1 and Figure 2, a strong positive linear relationship was observed between the Breastfeeding Self-efficacy and the LATCH breastfeeding success in obese and normal-weight mothers based on Pearson's correlation analysis (p<0.05, r: 0.613, r: 0.774).

**Table 3:** :Examination of relationship between BSES and LATCH breastfeeding success among obese and normal-weight mothers

	LATCH		
**Obese** **Mothers**	**BSES**	r p n	0.613*** **0.000** 113

**Normal-weight** **mothers**	**BSES**	r p n	0.774*** **0.000** 111

*: Breastfeeding Self-Efficacy Scale **: LATCH Breastfeeding Charting and Documentation Tool ***:p<0.01 r: Pearson's correlation analysis

In our study, the mean scores of LATCH breastfeeding success increased in line with an increase in the Breastfeeding Self-Efficacy Scale score in both obese and normal-weight mothers (p<0.05, r: 0.613). The relationship between BSES and LATCH breastfeeding success of obese and normal-weight mothers is given in Table 3, Figure 1 and 2.

The mean BSES score was 44.40±8.771 in obese mothers and 57.58±9.167 in normal-weight mothers. Accordingly, mean BSES score of normal-weight mothers was found to be significantly higher than that of obese mothers (p<0.001). As for the LATCH breastfeeding success scores, mean score was determined as 6.42 ± 1.301 in obese mothers and 8.62 ± 1.470 in normal-weight mothers. Mean scores obtained by obese and normal-weight mothers from the BSES and LATCH scales are given in Table 4.

**Table 4:** Comparison of mean scores obtained by obese and normal-weight mothers from BSES and LATCH scales

	Obese (n=113) X±SD	Non-obese (n=111) X±SD	t	p
**BSES**	44.40±8.771	57.58±9.167	0.994	0.000*

**LATCH**	6.42±1.301	8.62±1.470	1.853	0.000*

* *p<0.001*

t: Independent Samples T-Test

The mean scores of both Breastfeeding Self-Efficacy Scale and LATCH breastfeeding success were high in obese women who had information/training on breast milk in our study. Higher supplementary feeding rates in obese and normal-weight mothers were associated with lower mean scores of both Breastfeeding Self-Efficacy Scale and LATCH breastfeeding success. The total mean BSES and LATCH scores of obese and normal-weight mothers associated with having training on breast milk and supplementary feeding are given in Table 5.

**Table 5:** Comparison of total mean BSES and LATCH scores of obese and normal-weight mothers associated with having training on breast milk and supplementary feeding

			Having training		n	X±SD	t	p
**Obese** **mothers**	**BSES**	Yes			39	45.69±8.986	1.132	0.260
		No			74	43.73±8.641		
	**LATCH**	Yes			39	6.56±1.314	0.825	0.411
		No			74	6.35±1.297		
**Normal-weight** **mothers**	**BSES**	Yes			30	57.03±11.016	-0.385	0.701
		No			81	57.79±8.451		
	**LATCH**	Yes			30	8.67±1.516	0.171	0.864
		No			80	8.61±1.463		
**Obese** **mothers**	**BSES**	Yes			41	40.20±9.111	-4.11	0.000*
		No			41	46.81±7.650		
	**LATCH**	Yes			41	5.61±0.891		
		No			72	6.89±1.273	-5.684	0.000*
**Supplementary** **feedingn**		**X±SD**	**t**	**p**				
**Obese** **mothers**	**BSES**	Yes	41	40.20±9.111	-4.117	0.000*		
		No	72	46.81±7.650				
	**LATCH**	Yes			41	5.61±0.891		
		No			72	6.89±1.273	-	
					5.684	0.000*		
**Normal-weight** **mothers**	**BSES**	Yes	22	50.41±12.105	-4.435	0.000*		
		No	89	59.36±7.350				
	**LATCH**	Yes	22	7.41±1.532	-4.755	0.000*		
		No	89	8.93±1.294				

## Discussion

Breastfeeding self-efficacy of normal-weight mothers was found to be higher than that of obese mothers in our study (obese mothers: 44.40±8.771, normal-weight mothers: 57.58±9.167). Upon comparison of mean scores of the LATCH breastfeeding success, it was observed that obese mothers had a lower mean score than the non-obese . In Tokat's study where mothers were trained in the prenatal period, breastfeeding self-efficacy score in postnatal week 1 was found to be 56.3 ± 12.6 with a mean score of 7.46±1.00 obtained from the LATCH breastfeeding success 8. Kronborg et al. determined a relationship between maternal obesity and low maternal self-confidence in their study conducted on 1,597 Danish women 19. Kucukoglu's study on mothers of low-birth-weight infants determined pre-test total mean scores of breastfeeding self-efficacy scale as 41.77±11.40 and mean LATCH breastfeeding success as 5.81±1.72 among mothers in the control group 20. Several studies revealed that high breastfeeding self-efficacy level of mothers is very effective on maintenance of breastfeeding 21. There are some studies suggesting that obese mothers had low determination in meeting their breastfeeding goals and had more limited relatives, friends and social circle breastfeeding their babies 22. However, no study was found on breastfeeding self-efficacy levels of obese mothers in the literature.

Our study found determined no relationship between having training and mean BSES scores and mean score of LATCH breastfeeding success. Onbasi et al. studied prenatal breastfeeding training on breastfeeding behavior and found out that the use of water, pacifier, formula as well as exclusive breastfeeding rate in the first 6 months was higher in the trained group than the control group 23. Noel-Weiss et al. established that breastfeeding self-efficacy score after antenatal breastfeeding training was significantly high compared to the control group^24^. Chapman et al. found that breastfeeding education affected exclusive breastfeeding uptake up to postpartum 2 weeks in overweight and obese women 25. Our study revealed no statistically significant relationship between groups having training and no training and found high mean scores of BSES and LATCH breastfeeding success in trained obese mothers. This difference in this study was believed to result from the facts that breastfeeding trainings had conceptual frameworks in examined studies and that mothers who declared to have training underwent only standard, informatory training.

Higher supplementary feeding rates in obese and normal-weight mothers were associated with lower mean scores of both BSES and LATCH breastfeeding success in our study with a statistically significant difference. Baker et al. found that the rate of formula feeding increased with higher maternal BMI 26. Kronborg et al. found out that obese mothers gave formula at higher rates in their study on Danish women 19. In their study on 1,151 normal-weight and 580 obese mothers, O'Sullivan et al. established that exclusive breastfeeding rate was 43% in primiparous normal-weight mothers, but only 29% in obese mothers who continued breastfeeding 1 month postpartum. Exclusive breastfeeding rate was 57.1% in primiparous normal-weight mothers and 50.3% in obese mothers who continued breastfeeding in postpartum 2 months. Similar results were obtained in multiparaous normal-weight and obese mothers with fewer obese mothers who continued exclusive breastfeeding in 1 and 2 months than normal-weight mothers^27^. In their study examining the effect of maternal obesity on breastfeeding success on Hispanic and black women, Kugyelka et al. found no relationship between MSI and formula feeding in two groups; however, a higher rate of supplementary formula feeding was observed in obese Hispanic women 28. Bartok et al. identified a higher rate of formula feeding in the hospital stay among overweight and obese mothers 29. Mannion and Mansell conducted a pilot study on breastfeeding self-efficacy and use of galactagogue and found out higher use of formula with decreasing breastfeeding self-efficacy score 30. Our results are consistent with the literature. It is believed that these low rates result from low number of women who had breastfeeding training.

## Limitations of the study

Data obtained in this study was limited to self-declarations of mothers who delivered at Zekai Tahir Burak Hospital on the study dates.

## Conclusion

Our study revealed low self-efficacy and breastfeeding success in obese mothers compared to normal-weight mothers. A significant relationship was found between breastfeeding self-efficacy and breastfeeding success in both obese and normal-weight mothers. In the light of the data, and in order to increase the breastfeeding success among obese mothers, it is suggested that the perception of self-efficacy is enhanced, and additional consultation is provided and breastfeeding starting from the antenatal period. The training given to mothers by the midwife and nurse should be re-inforced with home visits especially in the obese women in the postnatal period in order for them to breastfeed.

## Authors' Note

• This study was presented as an Oral Presentation at the 1st International Congress on Women's Health and Education, Kocaeli-Turkey, 14–15 April 2016.

• This study was produced from the Master's thesis. Ankara Yıldırım Beyazıt University, Institute of Health Sciences, Master Thesis, 2016.
